# 肺癌家族聚集性的系统评价

**DOI:** 10.3779/j.issn.1009-3419.2010.03.07

**Published:** 2010-03-20

**Authors:** 俊东 谷, 峰 滑, 殿胜 钟, 军 陈, 红雨 刘, 清华 周

**Affiliations:** 300052 天津，天津医科大学总医院，天津市肺癌研究所，天津市肺癌转移与肿瘤微环境重点实验室 Tianjin Key Laboratory of Lung Cancer Metastasis and Tumor Microenviroment, Tianjin Lung Cancer Institute, Tianjin Medical University General Hospital, Tianjin 300052, China

**Keywords:** 肺肿瘤, 家族聚集, 系统评价, Lung neoplasms, Familial aggregation, Systematic review

## Abstract

**背景与目的:**

四十多年前Tokuhata和Lilienfeld首次从流行病学的角度证实肺癌的家族聚集现象。随后的多个研究也证实了家族聚集现象是肺癌危险性的一个家族性成分，但研究结果并不完全一致。本研究旨在进一步分析和评价一级亲属患肺癌者在肺癌发生中的作用。

**方法:**

检索PubMed、CENTRAL、中国生物医学文献数据库系统（CBM）、中国期刊全文数据库（CNKI）、中文科技期刊全文数据库（VIP）等收集国内外公开发表的肺癌家族聚集性的病例对照研究。采用RevMan version 4.2统计软件对各研究结果进行分析，计算其合并优势比和95%可信区间。

**结果:**

纳入合并分析的文献共有28篇，分析结果表明：肺癌先证家系一级亲属患肺癌的风险是对照家系一级亲属的1.88倍（*P* < 0.001)。分层分析，先证家系的父亲、母亲及兄弟姐妹患肺癌的风险分别是对照家系的1.62倍（*P* < 0.001）、1.96倍（*P* < 0.001）和1.92倍（*P* < 0.001）；吸烟和非吸烟先证者一级亲属患肺癌的风险分别是对照组中吸烟者和非吸烟者一级亲属患肺癌的1.73倍（*P* < 0.001）和1.42倍（*P*=0.02）；女性和男性肺癌先证者一级亲属患肺癌的风险性分别是对照组中女性和男性一级亲属患肺癌的1.89倍（*P* < 0.001）和1.99倍（*P* < 0.001）。

**结论:**

先证家系一级亲属患肺癌的风险性增加，肺癌存在明显的家族聚集现象。

## Abstract (en)

**Background and objective:**

Fourty years ago, Tokuhata and Lilienfeld provided the first epidemio- logic evidence of familial aggregation of lung cancer. Familial aggregation and increased familial risk for lung cancer have been reported in several studies, subsequently. But the results are not consistent with each other. The aim of this study is to further explore the relationship between family history of lung cancer and lung cancer risk.

**Methods:**

By searching PubMed, CENTRAL, CBM, CNKI and VIP, we collected both domestic and overseas published documents before November, 2009 on family history of lung cancer and lung cancer risk. RevMan version 4.2 was used to perform *meta*-analysis on the case-control study results, the combined odds ratio (OR) and the 95% confidence interval (CI) were calculated as well.

**Results:**

Twenty- eight publications were included into the combined analysis, which indicated that the lung cancer risk of the probands' first- degree relatives was 1.88 times higher than that of their controls' (*P* < 0.001). In the sub-study, compared with the controls' father mother and siblings, the OR of the probands' father mother and siblings was 1.62 (*P* < 0.001), 1.96 (*P* < 0.001) and 1.92 (*P* < 0.001), respectively. For smoking status, lung cancer risk in first-degree relatives of smoking probands was 1.73 (*P* < 0.001) times higher than that of their corresponding controls'. And for non-smoking subjects the lung cancer risk was 1.42 (*P*=0.02) times higher in proboands' first-degree relatives. For gender categories, lung cancer risk in first-degree relatives of female pro- bands was 1.89 (*P* < 0.001) times higher than that of their corresponding controls'. And for male subjects, the lung cancer risk was 1.99 (*P* < 0.001) times higher in proboands' first-degree relatives.

**Conclusion:**

Lung cancer risk was increased in probands' first-degree relatives, and obvious familial aggregation of lung cancer was observed in this study.

肺癌已经成为当前世界范围内的一个重要健康问题，每年大约有超过一百万人死于肺癌^[1]^。但肺癌发病的原因尚未完全明了，一般认为肺癌的发生是环境与遗传因素相互作用的结果。四十多年前Tokuhata和Lilienfeld^[2]^发现肺癌患者亲属中肺癌致死的人数高于对照组亲属，家族聚集现象是肺癌危险性的一个家族性成分。家族危险性表示为病例组较对照组更可能报告有肺癌的家族史。但关于肺癌家族聚集性的研究结果并不完全一致。本文收集2009年11月以前国内外公开发表的肺癌家族聚集性的病例对照研究进行系统评价，从循证医学的角度探讨肺癌家族史作为肺癌发生危险因素的作用。

## 1 资料与方法

### 1.1 资料来源

通过计算机检索PubMed、CENTRAL、中国生物医学文献数据库系统（CBM）、中国期刊全文数据库（CNKI）、中文科技期刊全文数据库（VIP）收集2009年11月以前国内外公开发表的肺癌家族聚集性的病例对照研究。

### 1.2 检索策略

英文数据库使用自由词检索，检索词包括：“lung cancer”、“lung neoplasm”、“neoplasm of the lung”；“risk”；“family history”；“familial aggregation”。检索语种为英语。中文数据库使用关键词检索，检索词包括：“肺癌”、“家族聚集”、“家族史”。为尽量避免漏查文献，对所检索文献中提供的参考文献进行二次检索，相关综述、会议摘要都被检索，以发现可能合格的研究。

### 1.3 纳入标准

① 发表时间为2009年11月以前公开发表的文献；②研究类型为病例对照研究；③研究对象为病理证实或临床诊断为肺癌患者；④各文献研究设计及研究方法相似；⑤原文献提供原始数据能够进行OR值及95%CI计算；⑥暴露及各因素分层划分标准基本相似。

### 1.4 排除标准

① 文献质量较差，未能提供原始数据用以计算OR值及95%CI者；②研究类型为非病例对照研究；③非中、英文文章。

### 1.5 文献筛选和资料提取

2位研究者独立阅读所获文献题目和摘要，在排除明显不符合纳入标准的文献后对可能符合纳入标准的文献阅读全文。由2位研究者交叉核对纳入研究的试验结果，对存在分歧的试验通过讨论或请教该领域的专家决定其是否纳入，最终确定纳入研究的文献。提取纳入文献的题目、作者、发表日期和文献来源、研究对象的基本情况以及各试验研究的基本数据。

### 1.6 质量控制

对所有符合纳入标准的文献进行质量评价，参照病例对照研究文献质量评价标准，对重复报告、质量较差、报道信息太少等无法利用的文献予以剔除。

### 1.7 统计分析

阅读文献，按照系统评价要求整理数据，建立数据库，并核校数据。用优势比（odds ratio, OR）来分析肺癌家族史与肺癌发生的关系。所有统计分析用统计软件RevMan version 4.2完成，纳入研究结果间的异质性采用*χ*^2^检验，如纳入研究结果不存在明显的异质性（*P* > 0.05），采用固定效应模型进行分析；反之，如果存在异质性（*P* < 0.05），则用DerSimonian-Laird法随机效应模型分析，并对可能导致异质性的因素进行亚组分析。采用倒漏斗图来评价发表偏倚。

## 2 结果

### 2.1 文献基本情况

纳入本次分析的28篇文献^[2-29]^的基本情况（表 1）。其中病例组16 936例，对照组21 741例，纳入研究的每篇文献均提供了病例组和对照组的原始数据。其中有13篇^[2, 4, 5, 9-12, 14-17, 21, 24]^文献提供了先证家系中双亲患肺癌的情况（表 2），研究结果间不存在明显的异质性（父亲：*χ*^2^=7.71，*P*=0.81；母亲：*χ*^2^=18.38，*P*=0.10）；11篇^[2, 4, 5, 9, 11, 12, 14-17, 19]^文献提供了先证家系中兄弟姐妹患肺癌的情况（表 2），研究结果间不存在明显异质性（*χ*^2^=13.76, *P*=0.18）；13篇^[2, 4, 5, 9, 11, 12, 16-18, 21, 22, 24, 26]^文献提供了非吸烟先证者一级亲属患肺癌的情况，研究结果间存在异质性（*χ*^2^=29.6, *P*=0.003）；10篇^[2, 4, 5, 9, 11, 17, 20, 22, 24, 26]^文献提供了吸烟先证者一级亲属患肺癌的情况，研究结果间不存在明显异质性（*χ*^2^=4.91, *P*=0.84）；14篇^[2, 5, 8, 11-13, 15, 19-23, 25, 26]^文献提供了女性肺癌先证者一级亲属患肺癌的情况，研究结果间不存在明显异质性（*χ*^2^=21.37, *P*=0.07）；5篇^[2, 13, 15, 19, 23]^文献提供了男性肺癌先证者一级亲属患肺癌的情况，研究结果间不存在明显异质性（*χ*^2^=8.27, *P*=0.08）。

**1:** 纳入研究文献的基本特征 General characteristics of included publication

First author	No. of cases	No. of controls	Study type	Sex	OR	95%CI	Years of data collection	Year of publicaion	Region
Tokuhata^[2]^	270	270	Case-control	M/F	3.87	1.88-7.99	1960-1961	1963	US
Cassidy^[3]^	693	1 299	Case-control	M/F	1.49	1.16-1.91	2002-2006	2009	Europe
Gao^[4]^	1 436	1 643	Case-control	M/F	1.73	1.43-2.10	2002-2005	2009	Italian
Rachtan^[5]^	1 058	2 116	Case-control	F	1.91	1.52-2.39	2004-2007	2009	Poland
Cassidy^[6]^	579	1 157	Case-control	M/F	1.21	0.94-1.55	1998-2004	2006	UK
Gorlova^[7]^	280	242	Case-control	M/F	0.82	0.50-1.34	1995-2003	2006	US
Neuberger^[8]^	413	614	Case-control	F	1.70	1.24-2.33	1994-1997	2006	US
Cote^[9]^	629	773	Case-control	M/F	2.19	1.59-3.03	1990-2003	2005	US
Jin^[10]^	740	740	Case-control	M/F	1.76	1.33-2.33	1992-1999	2005	China
Matakidou^[11]^	1 482	1 079	Case-control	F	1.60	1.25-2.05	1999-2004	2005	UK
Wu^[12]^	108	108	Case-control	F	4.35	1.79-10.55	1992-2002	2004	Taiwan
Jin^[13]^	370	370	Case-control	M/F	1.91	1.37-2.67	1987-1990	2002	China
Wunsch-Filho^[14]^	334	578	Case-control	M/F	1.76	0.78-3.96	1989-1991	2002	Brazil
Bromen^[15]^	945	983	Case-control	M/F	1.91	1.31-2.77	1988-1993	2000	Germany
Mayne^[16]^	437	437	Case-control	M/F	1.96	1.20-3.20	1982-1984	1999	US
Kreuzer^[17]^	2 226	2 302	Case-control	M/F	1.52	1.19-1.94	1990-1996	1998	Germany
Brownson^[18]^	618	1 402	Case-control	M/F	1.31	0.98-1.76	1986-1991	1997	US
Schwartz ^[19]^	257	277	Case-control	M/F	1.33	0.76-2.33	1984-1987	1996	US
Xiang^[20]^	649	675	Case-control	F	2.85	1.68-4.83	1992-1993	1996	China
Wu^[21]^	646	1 252	Case-control	F	1.35	0.95-1.93	1985-1988	1996	US
Osann^[22]^	208	208	Case-control	F	1.97	0.86-4.52	1969-1977	1991	US
Liu^[23]^	110	426	Case-control	M/F	3.22	1.82-5.70	1985-1986	1991	China
Shaw^[24]^	943	955	Case-control	M/F	1.75	1.31-2.32	1976-1980	1991	US
Wu^[25]^	334	335	Case-control	F	4.42	2.40-8.13	1983-1986	1988	US
Horwitz^[26]^	112	224	Case-control	F	2.38	0.84-6.75	1977-1982	1988	US
Guo^[27]^	205	200	Case-control	M/F	7.75	2.67-22.53	1981-1983	1987	China
Ooi^[28]^	336	307	Case-control	M/F	3.18	2.03-4.98	1976-1979	1986	US
Samet^[29]^	518	769	Case-control	M/F	3.30	1.84-5.95	1980-1982	1986	US
OR: odds ratio; F: female; M: male.									

**2:** 病例组与对照组中一级亲属父亲、母亲及兄弟姐妹患肺癌的百分比 Depiction of the profile of family history in first-degree relatives by father mother and siblings according to different authors

Authors\Relatives	Father case	Father control	Mother case	Mother control	Siblings case	Siblings control
Tokuhata^[2]^	1.9%, 5/270	0.4%, 1/270	1.9%, 5/270	0.7%, 2/270	2.2%, 25/1 152	0.7%, 7/1 002
Gao^[4]^	10.5%, 139/1 826	5.7%, 108/1 890	1.6%, 30/1 847	0.9%, 19/2 063	20.5%, 294/1 436	12.9%, 213/1 643
Rachtan^[5]^	8.9%, 95/1 058	5.2%, 110/2 116	2.2%, 23/1 058	1.1%, 23/2 116	6.3%, 67/1 058	3.1%, 66/2 116
Jin^[10]^	5.8%, 43/740	2.7%, 20/740	6.8%, 50/740	2.0%, 15/740	N.R.	N.R.
Matakidou^[11]^	8.7%, 120/1 382	5.5%, 57/1 030	3.3%, 43/1 305	2.0%, 20/993	6.6%, 89/1 351	3.8%, 38/1 011
Cote^[9]^	9.4%, 48/501	5.1%, 31/611	6.3%, 38/601	3.2%, 22/683	1.1%, 23/2 007	0.2%, 4/2 204
Wu^[12]^	6.5%, 7/108	2.8%, 3/108	12.9%, 14/108	1.9%, 2/108	4.6%, 5/108	1.9%, 2/108
Wunsch-Filho^[14]^	1.2%, 4/334	1.2%, 7/578	0.9%, 3/334	0.9%, 5/578	2.1%, 7/334	1.2%, 7/578
Bromen^[15]^	4.8%, 45/945	2.5%, 25/983	0.9%, 9/945	1.0%, 10/983	2.9%, 27/945	1.1%, 11/983
Mayne^[16]^	3.7%, 16/437	2.1%, 9/437	0.5%, 2/437	0.7%, 3/437	7.1%, 31/437	3.2%, 14/437
Kreuzer^[17]^	4.8%, 106/2 226	3.3%, 76/2 302	0.7%, 16/2 226	0.7%, 17/2 302	2.1%, 46/2 226	0.9%, 22/2 302
Wu^[21]^	3.4%, 16/464	2.4%, 30/1 252	1.9%, 9/464	0.5%, 6/1252	N.R.	N.R.
Schwartz^[19]^	N.R.	N.R.	N.R.	N.R.	6.2%, 16/257	6.9%, 19/277
Shaw^[24]^	4.3%, 41/943	3.1%, 30/955	1.6%, 15/943	0.7%, 7/955	N.R.	N.R.
N.R.: not report.						

**3:** 不同类别分层肺癌家族史与肺癌的关系 OR and 95% CI of lung cancer risk associated with family history of lung cancer in different categories

Categories	OR	95%CI	Z	*P*
Relatives affected				
Father^[2, 4, 5, 9-12, 14-17, 21, 24]^	1.62	1.43-1.82	7.85	< 0.001
Mother[2, 4, 5, 9, 12, 14, 17, 21, 24]	1.96	1.60-2.41	6.44	< 0.001
Siblings^[2, 4, 5, 9, 11, 12, 14-17, 19]^	1.92	1.68-2.19	9.58	< 0.001
Smoking status				
Smoking^[2, 4, 5, 9, 11, 17, 20, 22, 24, 26]^	1.73	1.54-1.94	9.48	< 0.001
Non-smoking^[2, 4, 5, 9, 11, 12, 16-18, 21, 22, 24, 26]^	1.42	1.06-1.91	2.34	0.02
Gender				
Female^[2, 5, 8, 11-13, 15, 19-23, 25, 26]^	1.89	1.68-2.12	10.69	< 0.001
Male^[2, 13, 15, 19, 23]^	1.99	1.52-2.61	4.98	< 0.001

### 2.2 统计分析结果

肺癌先证家系一级亲属患肺癌的风险是对照家系一级亲属的1.88倍（OR=1.88, 95%CI: 1.66-2.12）。总体检验效应，*Z*=10.17（*P* < 0.001）（图 1）。分层分析（表 3）可见，先证家系的父亲、母亲及兄弟姐妹患肺癌的风险分别是对照家系的1.62倍（*P* < 0.001）、1.96倍（*P* < 0.001）和1.92倍（*P* < 0.001）；吸烟和非吸烟先证者一级亲属患肺癌的风险分别是对照组中吸烟者和非吸烟者一级亲属患肺癌风险的1.73倍（*P* < 0.001）和1.42倍（*P*=0.02）；女性和男性肺癌先证者一级亲属患肺癌的风险性分别是对照组中女性和男性一级亲属患肺癌的1.89倍（*P* < 0.001）和1.99倍（*P* < 0.001）。

**1:**
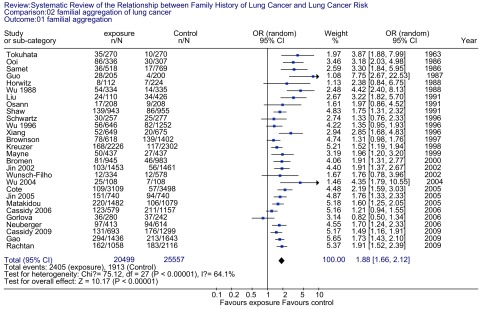
肺癌家族聚集性的森林图 Forrest plot of lung cancer risk associated with family history of lung cancer

### 2.3 发表性偏倚的识别

用统计软件RevMan version 4.2绘制倒漏斗图（图 2），图型基本对称，各点上呈漏斗状排列，说明发表性偏倚基本得到了控制。

**2:**
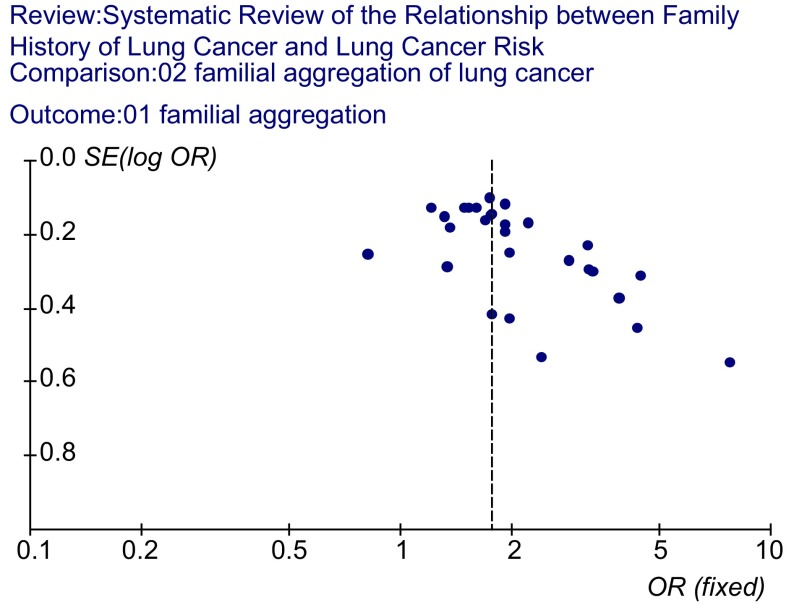
肺癌家族聚集性的倒漏斗图 Funnel plot of lung cancer risk associated with family history of lung cancer

## 3 讨论

现在已经明确，肺癌是一种环境相关疾病，大约有90%的肺癌均与烟草的暴露有关^[30]^。然而吸烟者中仅有10%-15%发生肺癌^[31]^，而有10%-15%的肺癌发生于非吸烟者中。显然，对肺癌致癌物的易感性存在个体差异，肺癌的发生并非完全由环境接触因素所决定。肺癌家族聚集现象的存在提示遗传易感性可能是一非环境接触因素。

某种疾病的家族聚集性是提示存在一个致病遗传组分的第一个证据。本系统评价证实先证家系一级亲属患肺癌的风险明显增加，为对照组的1.88倍。但是不能单纯的把1.88倍的肺癌发生风险完全归因于恶性肿瘤家族史，因为除了恶性肿瘤家族史外其他因素如共同的生活环境、家系结构等在肺癌的发生中也可能起到了作用，而且在进行病例对照研究时这些作用不太可能通过匹配的方法达到完全消除。另外在研究肺癌家族聚集性时吸烟是需要重点考虑的一个因素，因为吸烟在肺癌发生中的作用要大于肺癌家族史本身，因此在系统评价中我们对吸烟这个因素进行了分层分析，非吸烟先证者一级亲属患肺癌的风险是对照组的1.42倍；而吸烟先证者一级亲属患肺癌的风险是对照组的1.73倍，较非吸烟者高。吸烟和家族史相互作用，使有双重特质的人群（家族史阳性的吸烟者）具有高度的肺癌风险性。分层分析的结果显示先证家系中双亲患肺癌的风险性明显增加，该分层分析的结果稳定性较好，各研究结果间不存在明显异质性，同时每个病例只有一对双亲，不受家系结构的影响；先证家系中兄弟姐妹患肺癌的风险是对照组一级亲属的1.92倍，虽然该层次研究结果也不存在明显异质性，但其受家系结构（不同家系中兄弟姐妹的个数不同）的影响较为严重，其结果的稳定性可能受到一定的影响；女性和男性肺癌先证者一级亲属患肺癌的风险性分别是对照组中女性和男性一级亲属的1.89倍和1.99倍。提示先证家系中男性亲属可能更容易受肺癌家族史的影响，但由于家系结构的影响，其结果稳定性也受到影响。在不考虑家系结构对结果稳定性影响的情况下，肺癌先证家系中男性亲属患肺癌的风险性最高（为对照组的1.99倍），原因可能为肺癌先证家系中男性亲属吸烟者较多，该吸烟因素增加了患肺癌的风险。

分析肺癌家族聚集性的原因可能有以下几种解释：①造成肺癌危险的一个遗传组分；②家族成员中共有的环境危险因素如吸烟等；③影响家族危险因素特征的家族结果（如家系大小和年龄构成）在病例组和对照组不同。该研究对上述造成肺癌家族聚集性的原因进行了分层分析，分析结果在平衡环境因素和家系结构的影响后提示造成肺癌一个遗传组分的存在。

通过该项研究我们可以看到，除了环境接触因素外遗传因素在肺癌发生过程中起到了一定的作用，但遗传因素是如何发生作用的呢？一般认为，慢性病（如肿瘤）的遗传属于多基因遗传，肺癌的遗传并非肺癌的本身，而是个体对肺癌的遗传易感性。它的遗传方式并不符合孟德尔单基因遗传的遗传规律，而是属于多基因遗传（多因子遗传），即多个微小效应的基因，在某个或某些环境因子的作用下产生一个总效应从而导致肿瘤的发生^[32]^。但是，要想证实肺癌是一个遗传性疾病最直接的证据是找出导致肺癌发生的突变基因。那么在肺癌的遗传方式中是否存在一个决定肺癌遗传的主基因呢？近来的一项研究^[33]^发现肺癌中可能存在这样的一个基因座，它在肺癌发生的遗传因素中起了重要的作用，但还需要更多的研究结论予以证实。

从病例对照研究的角度来看，进行肺癌家族聚集性的研究存在一定的优势，该方面的研究基本可以消除回忆性偏倚。因为对研究人群进行调查时，对于恶性肿瘤家族史有无的回答往往比较肯定，同时也可以查询当地肿瘤发病登记处的登记得以证实。回忆性偏倚是影响病例对照研究结果的一个重要因素，该方面的研究基本消除了回忆性偏倚，使得系统评价的结论更为可靠。该系统评价也存在一定的缺陷，即未分层分析前各研究之间存在明显的异质性，尽管这种异质性可以通过统计学方法加以修正，但是它对于结论的影响程度是很难估计的，而且研究之间的异质性也不大可能通过因素调整达到完全消除。
